# Toluene Toxicity in the Brain: From Cellular Targets to Molecular Mechanisms

**DOI:** 10.1146/annurev-pharmtox-012924-010532

**Published:** 2025-01

**Authors:** Andrew A. Shaw, Jeffery D. Steketee, Anna N. Bukiya, Alex M. Dopico

**Affiliations:** Department of Pharmacology, Addiction Science, and Toxicology, College of Medicine, The University of Tennessee Health Science Center, Memphis, Tennessee, USA

**Keywords:** toluene brain intoxication, neurons, glia, brain vessels, membrane lipids, protein receptors

## Abstract

Toluene intoxication constitutes a persistent public health problem worldwide. While most organs can be damaged, the brain is a primary target whether exposure is accidental, occupational, or recreational. Interventions to prevent/revert brain damage by toluene are curtailed by the scarce information on the molecular targets and mechanisms mediating toluene’s brain toxicity and the common exposure to other neurotoxins and/or coexistence of neurological/psychiatric disorders. We examine (*a*) the physicochemical properties of toluene that allow this inhalant to primarily target the lipid-rich brain; (*b*) the cell types targeted by toluene (neurons, different types of glia), while considering a cerebrovascular component; and (*c*) putative molecular mechanisms by which toluene may modify receptor function while analyzing structural features that allow toluene to directly interact with membrane lipids or specific proteins. This information constitutes a stepping stone to design pharmacotherapies that counteract toluene brain intoxication.

## INTRODUCTION

Human exposure to organic solvents, including toluene, whether accidental, professional, or recreational, constitutes a resilient public health problem worldwide, with these chemicals particularly targeting vulnerable individuals with compromised access to health care and education (1–5). Moreover, the atmospheric levels of toluene and related organic solvents are expected to rise (6), and the increase in fracking industries leads to produced water that is contaminated by toluene. Produced water may be in agricultural areas, furthering human exposure to toluene (7). Lastly, abuse of inhalants [i.e., any volatile, noncombusted substance that is inhaled to experience a high and/or hallucinations (8, 9)] is associated with an increased risk for misuse of other psychotropic drugs, polysubstance use disorders (10–12), and serious psychological, neurological, and systemic impairments, even causing coma and death (13, 14). Remarkably, there is neither an antidote for nor a drug antagonist against toluene. Thus, treatment of toluene intoxication is limited to palliative measures, including support of central nervous system (CNS), respiratory, and cardiovascular functions (15). This limitation stems from the fact that the actual receptors and molecular mechanisms that drive the toxic actions of toluene remain largely unidentified. So far, significant advances have been made in identifying ionotropic receptors and the neurocircuitry involved in the reinforcing properties of toluene (for a recent review, see 16). However, much less is known about the cellular targets, subcellular processes, and molecular targets and mechanisms that participate in toluene’s brain intoxication. We aim at covering this gap of knowledge.

## TOLUENE CHEMISTRY AND THE BRAIN AS ORGAN TARGET

**Figure 1:**
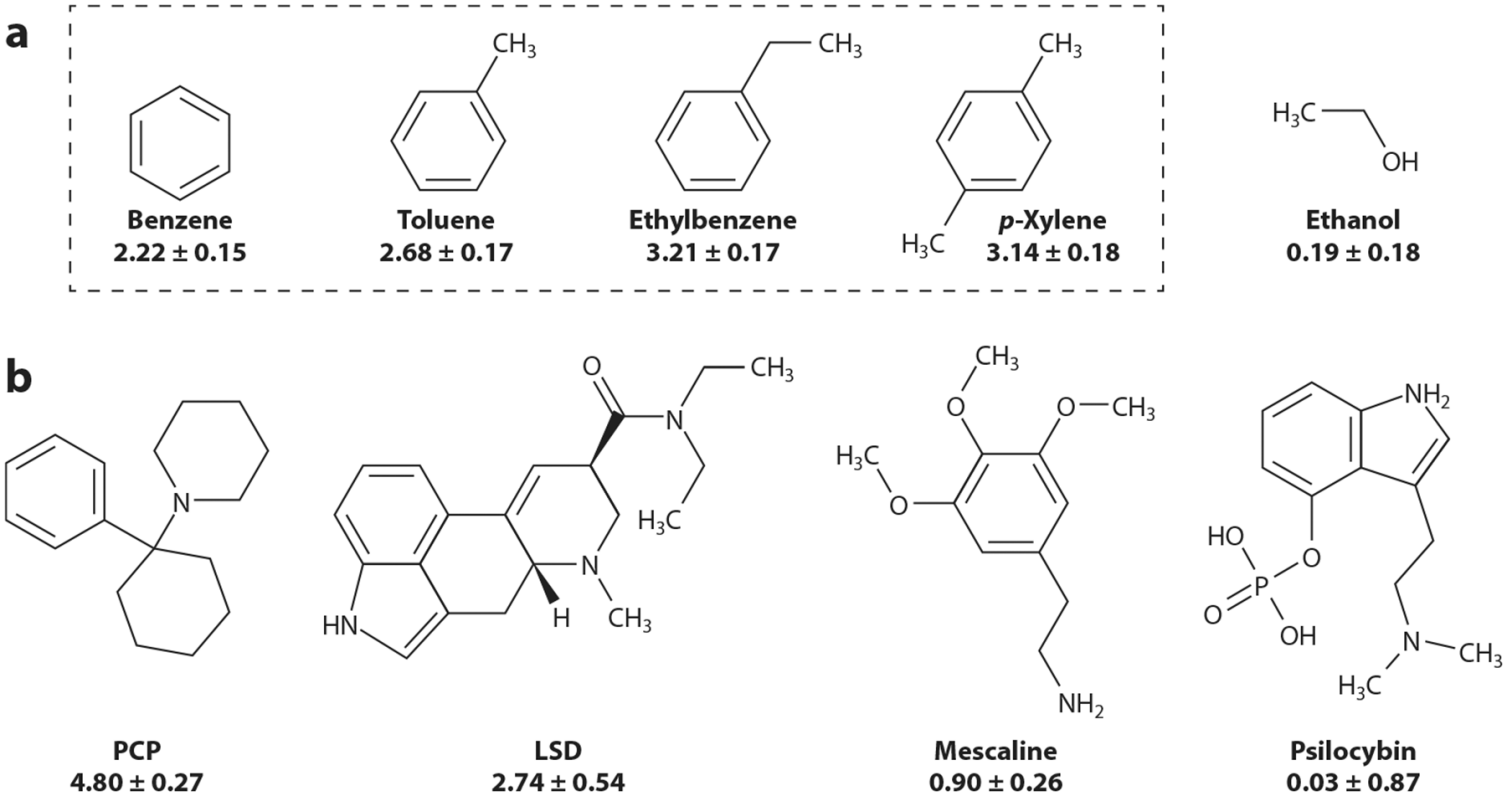
Brain-targeting organic compounds that may evoke hallucinations. (*a*) The benzene, toluene, ethylbenzene, and *p*-xylene (BTEX) compounds are shown inside a dotted box. The set is defined by simple chemical structures and targets of drug action that are not fully resolved. (*b*) Hallucinogens thought to cause their mind-altering effects through interaction with specific brain proteins, such as 5-HT receptors: phencyclidine or phenylcyclohexyl piperidine (PCP), lysergic acid diethylamide (LSD), mescaline (3,4,5-trimethoxyphenethylamine), and psilocybin (3-[2-(dimethylamino)ethyl]-1*H*-indol-4-yl dihydrogen phosphate). All structures were generated from their respective simplified molecular-input line-entry system (SMILES) using ACD/3D Viewer (ACDLabs). The number underneath each structure provides an index of the molecule hydrophobicity (logP). These values were predicted using a built-in function in ACD/3D Viewer. Overall, the hydrophobicity of BTEX compounds is only surpassed by PCP and matched by LSD, while greatly exceeding those of ethanol, mescaline, and psilocybin.

Despite the paucity of information on mechanisms and targets of drug action, there is little doubt that the brain constitutes the main organ targeted by toluene intoxication, whether from accidental, occupational, or recreational exposure, a fact that can be explained by the physicochemical properties of this volatile. Toluene is a benzene with a single methyl group (Figure 1a) and thus also known as methyl benzene, methyl benzol, phenyl methane, and toluol (15). Toluene and benzene, in conjunction with ethylbenzene and *p*-xylene, constitute the BTEX compounds (Figure 1a). When compared with other abused psychotropic drugs that are known to act upon specific protein receptors in the brain (Figure 1b), BTEX inhalants show remarkably simpler chemical structures and, on average, higher hydrophobicity (Figure 1). This property allows them to easily cross cell membranes, distribute throughout the body, and target lipid-rich organs such as the brain, where they may accumulate (17–20).

Toluene’s high hydrophobicity has been consistently linked to its ability to preferentially target myelinated structures of the brain and thus cause CNS toxicity (4, 15). The connection between toluene lipophilicity and brain damage is so strong that it led to speculation that pure toluene is more damaging to brain white matter than solvents containing additional toxic chemicals (e.g., acetates in paint thinner) (21). Lastly, the neurological events triggered by inhalation of toluene in humans or oral exposure in rodents are directly related to the concentrations of toluene in the brain. While toluene levels in alveolar breath have been proven to be an accurate and noninvasive indicator of the absorbed dose (22), other biomarkers (e.g., hippuric acid and other metabolites in urine) show lack of specificity and/or poor correlation with toluene exposure, particularly under occupational exposures (23, 24).

The primary role of white matter damage in toluene’s brain intoxication is buttressed by the very robust relationship between the duration of toluene abuse and metabolic abnormalities reflecting axonopathology and demyelination in the cerebellar and periventricular white matter regions (25). Moreover, the cognitive decline characteristic of chronic toluene users parallels the extent of white matter damage shown in neuroimaging (20, 26). Autopsies of toluene abusers confirm that cerebral and cerebellar white matter are primarily affected, often coexisting with normal cortical gray matter, and the sparing of axons within white matter until late in intoxication (27, 28). Imaging abnormalities are best characterized by abnormal myelinization, hyperintensities, callosal thinning, and loss of gray matter–white matter boundaries (21). However, Aydin et al. (25) underscore that axonal damage, rather than demyelination, is the basis of the white matter pathophysiology triggered by exposure to high toluene levels.

A systematic review of 30 studies involving chronic toluene abusers unveils a plethora of neurological deficits, the most common being reduced speed of information processing, coordination, attention, learning, short-term memory, executive ability, insight, and judgment (20, 21). This variety has been interpreted as characteristic of a noxa that overwhelmingly affects white matter (13, 29). Although white matter abnormalities usually occur earlier and are more pronounced, caution should be taken in ruling out gray matter compromise (30). In general, chronic toluene abusers do have reduced gray matter in various regions (31). Thus, in most people, long-term toluene abuse causes a chronic encephalopathy termed toluene leukoencephalopathy (TLE) that involves diffuse atrophy of the cerebral cortex, cerebellum, and brainstem, accompanied by sulcal widening and ventricular dilation (13, 17, 20, 21). At the cellular level, TLE consists of severe loss of myelin and a mild loss of axons themselves (17, 32). The role of different brain cell types in toluene’s brain intoxication is discussed next.

## TARGET CELLS

The cellular targets of toluene in the brain following occupational exposure in humans remain largely unknown (6). Most studies conducted in animal models or in vitro to get insight into cellular and molecular targets of toluene have employed experimental designs and toluene concentrations that match those found in heavily intoxicated humans. Such material, dealing with neurons, glia, and cerebrovascular components, is presented below.

### Neurons

Some brain regions are highly susceptible to toluene-induced injury, raising the hypothesis that specific neurons may be more vulnerable than others. Data from rats demonstrate that chronic toluene causes robust hippocampal CA1 histological and functional disruption (33–35), yet whether pyramidal neurons versus other neuronal types are differentially affected by toluene remains unanswered. In general, neurons are more sensitive to toluene in the developing brain, including adolescence; toluene may alter presynaptic modulation of CA1 pyramidal neurons through functional changes in *N*-methyl-d-aspartate (NMDA) receptors, which is significant during development (34). Since hippocampal CA1 neuron activity overwhelmingly contributes to memory formation and retrieval, the high vulnerability of these neurons to toluene could provide a basis for the memory and learning difficulties experienced by toluene abusers (21, 36). Notably, neuronal loss by chronic toluene is most pronounced in the second, third, and sixth layers of the parietal cortex and the cerebellar Purkinje cell layer (27).

Quantitative magnetic resonance spectroscopy data from chronic toluene users show a significant reduction in *N*-acetylaspartate (NAA) in the cerebellum and centrum semiovale, yet not in the thalamus (25). NAA reflects neuronal density and functional viability and has a higher concentration in the CNS white matter (37, 38). In agreement with Aydin et al. (25), such differential outcomes can be interpreted as toluene causing axonopathy in the absence of direct neuronal injury. This interpretation is also supported by animal studies showing that exposure to toluene inhalation for 6 months does not cause any change in the number of neurons (39). Likewise, human autopsies from long-term toluene abusers show, despite marked demyelination, little or no neuronal loss/damage (13, 27). While diffuse, ill-defined myelin pallor and discoloration of white matter are found in both cerebrum and cerebellum, these findings are considered to reflect axonal loss, also favoring axonopathy over myelin loss itself as the primary pathophysiological process driving toluene toxicity in neurons (27, 30). Consistently, choline levels are barely modified in chronic toluene abusers, which indicates that active demyelination is not the primary pathophysiological event (25). Collectively, we interpret that chronic toluene exposure does not exert an overall toxic effect on the neurons leading to cell death, but causes an axonopathology (21), with demyelination being an accompanying, likely secondary, event. Unfortunately, the mechanisms of this axonopathology remain unresolved. Toluene-induced white matter injury might cause impaired axonal iron transport with consequent gradual accumulation of iron in the cortex and deep gray matter. This hypothesis needs confirmation by histological analysis. Notably, other neurodegenerative disorders with similar radiologic findings due to axonal disruption of iron transport show iron deposition on Prussian blue staining (40, 41). Toluene might directly interfere with neuronal protein function secondary to increased oxidative stress and intracellular Ca^2+^ overload (15). Consistently, antioxidant injection to rats that had previously inhaled high levels of toluene for 12 days was able to reverse the reduced dendrite growth in layer II and III pyramidal neurons in the cerebral cortex and the blunted branching caused by toluene (42). Moreover, the hippocampus, whose functions are drastically impaired by toluene, is especially vulnerable to toluene oxidative stress (43).

### Glia Types

Collectively, all types of glial cells have a heightened sensitivity to neurotoxic damage, sustaining morphological alterations at lower concentrations than those required to morphologically alter neurons, which is considered an adaptation to protect neurons (44). Metabolic changes, however, may be found in the absence of morphological modification. Thus, myoinositol-containing compounds are an index of glial metabolism (45), and their increased levels in the cerebral and cerebellar white matter of toluene abusers may be indicative of reactive gliosis rather than glial loss (25). According to Demir et al. (46), reactive gliosis and astrocyte activation are the main causes of toluene-induced CNS damage. Microglia are the first responders after CNS injury as they have a higher sensitivity and play a role in coordinating reactive gliosis, which is primarily carried out by astrocytes. This astrogliosis serves the ultimate goal of protecting neurons and their functions through mechanisms such as blood-brain barrier restoration. However, astrogliosis may lead to damage of both neurons and oligodendrocytes via production of proinflammatory and cytotoxic cytokines (47). During reactive gliosis, intermediate filament proteins are upregulated, particularly glial fibrillary acidic protein (GFAP) (48). In mice, acute exposure to 2,000 and 4,000 ppm toluene evokes a significant increase in GFAP expression, with exposure to 1,000 ppm approaching statistical significance (49). Likewise, exposure to 1,500 ppm toluene for 4 h/day for 4–10 days increases astrocyte activation, gliosis, and GFAP expression in rat cerebellum and hippocampus (50). Following a chronic model of toluene exposure (2,000 ppm for 4 h/day for a month), large increases in the density and area of GFAP-immunoreactive processes in astrocytes of adult rats are found, indicating increased astrocyte activation (51). As suggested by several studies (51, 52), increased astrocyte and microglial activity due to toluene exposure, whether acute or chronic, could be part of a neuroinflammatory response that disrupts neurotransmitter levels. Indeed, upon activation, microglia trigger the activation of astrocytes through the secretion of cytokines IL-1α, tumor necrosis factor-α (TNF-α), and complement component 1q (C1q) (53). Activated astrocytes eventually cause neuronal and glial injury by producing proinflammatory factors such as reactive oxygen species, TNF-α, and IL-1β, which damage the CNS (54). This may occur through synaptic tagging, in which astrocytes release factors that allow microglia to eliminate synapses (55). Exposure to 1,500 ppm toluene for 4 h/day for 7 days causes an overexpression of transforming growth factor-β1 (TGF-β1) in rat blood vessel endothelium (56). Rats exposed to 650 mg/kg toluene for 15–45 days show increased caspase-3, another apoptotic marker (57). In addition to activated expression of nicotinamide adenine dinucleotide phosphate oxidase by microglia, which results in the production of superoxide and reactive oxygen species, there is a marked increase in the levels of inducible nitric oxidase. Thus, conversion of arginine into nitric oxide exacerbates the excitotoxicity of glutamate (55).

Chronic activation of microglia and astrocytes is known to facilitate the development of neurodegenerative diseases such as Alzheimer’s disease (AD) and Parkinson’s disease (53, 58). Remarkably, chronic toluene abusers are commonly reported to exhibit AD, other forms of dementia, and Parkinson’s disease–like symptoms (59). Thus, aside from modifications in dopaminergic (DA) and cholinergic neurotransmission (60, 61), the neurodegenerative disease signs and symptoms of toluene abusers could be due to microglia-astrocyte overactivity and eventual neuroinflammatory and neurotoxic processes.

Regarding possible molecular mechanisms underlying toluene-induced neurotoxicity, NMDA antagonism has been shown to induce neurodegeneration in developing rat neurons (62). Since toluene has been shown to inhibit in a concentration-dependent manner recombinant NMDA receptor subtypes found in the brain (63), it is possible that toluene could induce neurodegeneration as reported for other NMDA antagonists.

Oligodendrocytes, in turn, support neurons by forming the myelin sheaths around axons, which allow for faster (saltatory) conduction (64). Toluene damages myelin sheaths in humans, yet the precise threshold of exposure that induces demyelination is unclear, as most studies examine patients who suffer from other conditions favoring demyelination (13). Actual damage to the myelin sheath with eventual degenerative changes resulting in either shrinking or swelling of axons has been reported, albeit for Schwann cells, in rats exposed to 3,000 ppm toluene for 8 h/day, 6 days per week for 16 weeks (65).

Generally, before myelin damage can be detected, glial damage occurs, usually evidenced by astrogliosis and the presence of perivascular Periodic acid–Schiff-positive macrophages that contain laminar or coarse myelin debris (13). In addition to the mediation of demyelination via removal of myelin debris, microglia can induce demyelination through their own activation, the release of inflammatory cytokines, and astrocyte activation. The latter amplifies, rather than initiates, demyelination (66). The relative contribution of these processes and glial types to toluene-induced demyelination remains to be established.

Lastly, detailed cytological analysis of toluene-induced injury of oligodendrocytes has been recorded from adult rabbits acutely administered 876 mg/kg of toluene: Irregular morphological changes in the nucleus structure, pyknosis, necrosis, vacuolar degeneration, and gliosis have all been reported (46), although the molecular underpinnings remain unknown.

### Cerebrovascular Component

The lack of overt inflammatory and/or vascular alterations in most patients who chronically abused toluene has been claimed as a useful criterion to distinguish TLE from demyelinative or immune-mediated neuropathology and from cerebrovascular diseases (13). However, several considerations strongly suggest the involvement of a neurovascular component in toluene-induced disruption of brain function, particularly during acute intoxication. First, the most richly perfused organs, which are also highly vulnerable to ischemia, such as kidneys, heart, and brain, are severely damaged by toluene (67). Sudden sniffing death syndrome was initially defined as sudden and unexpected death by solvent inhalation without a plastic bag causing suffocation (68). Of note, sudden sniffing death from toluene inhalation includes cardiac malfunction, which compromises proper blood supply to the brain, and CNS depression as major mechanisms leading to death (69). Second, the neural symptoms/signs of acute intoxication by toluene almost totally overlap with those of cerebral ischemia (70, 71): Decreased/loss of consciousness, confusion/disorientation/impaired cognition and intellect, changes in vision/visual perception, dizziness/vertigo, impairment of speech/hearing, motor incoordination, drowsiness, and headaches are frequently found in both conditions. Third, aside from the increased risk of cranial trauma associated with the behavioral effects of drugs of abuse, including toluene, the neurological complications resulting from drug misuse are basically driven by cerebrovascular events (72). Fourth, frequent toluene use during a 3-year-long period causes involuntary emotional expression disorder, which includes laughing and crying without emotions of happiness or sadness. Single-photon emission computed tomography data in a subject suffering from this disorder due to toluene abuse show left frontoparietal hypoperfusion (73). Likewise, semiquantitative positron emission tomography (PET) data show that chronic solvent abusers present a bilateral decrease in blood flow, which is particularly significant in the prefrontal cortex (PFC) (74). These authors speculated that the reduced PFC blood flow may be responsible for the amotivational syndrome seen in these patients. PET data from chronic toluene users unveil perfusion deficits in not only the PFC but also parietal and temporal regions (74, 75). Collectively, brain hypoperfusion appears to be present in more than 70% of chronic toluene abusers (21). Lastly, initial data from our group demonstrate that toluene at concentrations reached in blood during inhalation for recreational purposes evokes a reversible and concentration-dependent constriction of rat middle cerebral arteries whether toluene is inhaled (76) or applied in vitro to isolated brain artery segments (77). The latter suggests that systemic, neuronal, or glial factors are not critical for this toluene action.

Notably, magnetic resonance imaging data from an individual with an 8-month history of solvent abuse do not show any of the gross anatomical abnormalities found in 5-year-long users but point at multifocal hypoperfusion in areas consistent with frontal and subcortical abnormalities (78). The differential outcomes of month- versus years-long toluene users led to the speculation that perfusion deficits may be an early sign of neurobiological changes (axonal injury and/or demyelination), predating deficits detectable with structural imaging (21).

## MOLECULAR TARGETS

### Ionotropic Receptors

With the goal of investigating the addictive/reinforcing properties of toluene, researchers have clearly documented that this volatile, similar to other drugs of misuse, is able to interact with specific neurotransmitter systems: largely DA, γ-aminobutyric acid (GABA)ergic, and glutamatergic components of the ventral tegmental area (VTA), caudate, nucleus accumbens (NAc), and PFC (3, 16, 33, 60, 79, 80). This body of evidence strongly suggests that toluene affects some determinants of neuronal excitability (e.g., specific ion channel populations) and not others. Moreover, the first theme that emerges from studies examining toluene functional interactions with ionotropic receptors is that toluene not only targets several ion channel populations within the same cell but also exerts differential actions on one channel subfamily versus another. For example, toluene applied acutely to rat hippocampal neurons strongly and reversibly decreases NMDA-mediated currents (IC_100_ ~ 10 mmol = 1,064 ppm) while, at the same concentrations, it fails to modify α-amino-3-hydroxy-5-methyl-4-isoxazolepropionic acid (AMPA)-mediated currents and barely increases kainate-mediated currents (81). In the same species, toluene also reduces NMDA-mediated excitatory postsynaptic currents (EPSCs) and fails to alter AMPA-currents in PFC layer II neurons. However, AMPA-mediated EPSCs are delayed, an effect prevented by pharmacological block of internal calcium mobilization (82). The differential pharmacology of toluene on variant channels that are naturally activated by glutamate has been validated on recombinant channels that are expressed heterologously. Thus, the differential actions of toluene on ion channels of known subunit composition and primary sequence when expressed in the same membrane and under identical recording conditions have advanced the idea that a direct toluene–ion channel protein interaction or a direct interaction between toluene and a third, nearby party (channel-associated membrane protein, specific membrane lipid species in the vicinity of the ion channel) that is differentially recognized by channel subunit/distinct amino acids mediates toluene action. Indeed, acute toluene exposure evokes an increase in GluA6-containing AMPA activity, has no effect on GluA1 or GluA1/2 AMPA subtypes, and dose-dependently and subunit-selectively inhibits NMDA current following expression in *Xenopus laevis* oocytes (63). Thus, it has been proposed that glutamate ionotropic receptor subunits contain discrete sites that distinctly recognize toluene molecules (63, 83). Toluene action, however, is shared with other highly lipophilic BTEX members of different chemical structure (84) (Figure 1a). Second, toluene antagonism of NMDA and glycine (Gly) receptors remains even when channels are exposed to high agonist concentrations, which suggests a noncompetitive antagonism (63). Third, toluene (albeit >20 mmol) was reported to increase ion leak through membranes (63, 83). Therefore, it cannot be totally ruled out that acute actions of toluene (especially at very high concentrations) on glutamate-gated channels could involve direct interactions with membrane lipids and/or an allosteric, or with a very low affinity hydrophobic site(s) in a membrane protein (even the channel itself).

Regarding protracted exposures, 4-day toluene application to rat hippocampal neurons enhances NMDA-mediated currents without affecting the acute sensitivity of these channels to toluene. Kainate and AMPA currents remain unaffected. Consistently, the amplitude of NMDA EPSCs is increased by toluene in the absence of changes in AMPA EPSCs (81). Thus, chronic toluene exposure exerts a differential effect on glutamate ion channels that qualitatively matches the outcome observed after acute applications. Toluene actions on ionic currents are accompanied by protein expression changes: Immunoblot data show a drastic upregulation of GluNR2A and GluNR2B subunits with no change in NR1 subunits. NR1 subunit density, however, is increased (81). Remarkably, changes in subunit composition due to chronic toluene exposure are brain region specific: Western-blotting data show that a 10-day-long toluene exposure increases GluN1, GluN2B, and GluA2/3 in the rat medial PFC, barely increases GluN2B in NAc, and decreases GluN1 in the substantia nigra (85).

When considering connections between in vitro findings and behaviors altered by toluene, drug actions on glutamate-driven EPSCs and inhibitory postsynaptic currents (IPSCs) in rat PFC slices have been proposed as ionic bases of toluene’s detrimental effects on cognition and decision making (82). In this and all other in vitro studies, caution should be exerted: Identified mechanism(s) should operate similarly in vivo to explain a given toluene effect in the body. Thus, toluene evaluation upon gene manipulation of putative target(s) in the whole organism is needed.

In rat hippocampal neurons, acute toluene also inhibits nicotinic acetylcholine receptor (nAChR) currents in a concentration-dependent and reversible manner (IC_50_ = 1.1 mmol) (61, 83). When expressed in *X. laevis* oocytes, both human and rat α4/β2 nAChR-mediated currents are inhibited by more than 80% in response to 0.9 mmol of toluene, with IC_50_s being in the same range (0.15–0.9 mmol) (61, 83). Toluene’s efficacy and potency on human and rat α3/β2 channels are identical (~60% inhibition and IC_50_ = 0.13–0.18 mmol). Lastly, acetylcholine-preactivated human and rat α7 nAChRs are both inhibited by 0.3 mmol toluene with very slight differences in efficacy and potency between species. Collectively, there are no major differences in the toluene pharmacology of these three nAChRs between species. Regarding the rat constructs, however, α4β2 and α3β2 are significantly more sensitive to toluene than α4β4, α3β4, and α7 (61). Notably, reciprocal point substitutions in the heteromeric channels (β2V253F and β4F255V) introduce responses that match those of the intended mutation (β4 and β2, respectively), unveiling the possibility of specific toluene sensing by distinct residues in nAChRs. These studies also underscore that two out of the three nAchRs most common in the CNS (α4β2, α3β2, and α7) are more sensitive to toluene than their peripheral nervous system (PNS) counterparts.

Regarding inhibitory neurotransmitters, acute treatment of rat hippocampal CA1 or PFC neurons with less than 1 to 3 mmol toluene significantly increases GABA_A_-mediated IPSCs (82, 86). In the former, toluene rather selectively enhances the frequency of GABA_A Slow_ miniature inhibitory postsynaptic currents (mIPSCs) and the number of slow events that contribute to the mIPSCs; toluene action on inhibitory transmission may be presynaptic (increased GABA release), without major effects on intrinsic (postsynaptic) excitability. Indeed, toluene action persists after blocking action potential (AP) and/or sodium channels. Consistently, toluene has no discernible effect on AP peak amplitude, decay phase, half-width, duration, or rise time (87). However, as found for toluene action on glutamate-driven currents, toluene-evoked increases in mIPSC frequency are blunted by ryanodine or dantrolene, yet not thapsigargin, suggesting that toluene induces release of Ca^2+^ from caffeine- but not IP_3_-sensitive stores (87). Whether these Ca^2+^-mediated events operate independently of toluene-GABA cross-talking at the receptor itself remains unknown.

When considering protracted exposures, treatment of hippocampal neurons with toluene for 4 days causes a significant reduction in the amplitude of GABA-mediated currents (81). Western blotting, however, shows increased GABA_A_ α1 subunits in the medial PFC and a decrease in the substantia nigra. In turn, mild increases and small decreases in GABA_A_ α1 have been found in the striatum and VTA (85). Collectively, toluene-induced potentiation of GABA-mediated inhibition and reduction of NMDA action in rat CA1 hippocampal slices has been claimed to explain the cognitive impairment reported in inhalant abusers (87).

Following expression in *X. laevis* oocytes, acute toluene increases both GABA α1β1 and Glyα1 receptor activity. In both, toluene action is lost in the absence of a respective agonist (GABA, Gly), suggesting that toluene’s functional interaction with these receptors is allosteric (33, 86). Likewise, type 3 serotonin receptors (5-HT_3_), which belong to the same channel family as GABA_A_ and Gly, increase activity in response to submillimolar and millimolar doses of toluene only in the presence of their natural ligand serotonin (5-HT) and fail to do so in its absence (88).

Within the extracellular ligand-gated ion channel family, we lastly consider a group of ATP-gated ion channels (P2X), the subtypes commonly found in the CNS being P2X_2_, P2X_4_, and P2X_6_. After expression in HEK293 cells, P2X_2_, P2X_4_, P2X_2/3_, and P2X_4/6_ render ATP-evoked inward currents that are reversibly and dose-dependently potentiated by submillimolar and millimolar doses of toluene. As found with GABA-, Gly-, and 5-HT-gated channels, toluene became inefficacious in the absence of the natural ligand (i.e., ATP). Toluene also failed to introduce a significant rise over the channel maximal response to ATP, suggestive of allosteric modulation. In contrast, toluene inhibits recombinant P2X_3_, which is found primarily in the PNS (89). It is likely that the differential response to toluene from P2Xs that are abundant in the PNS versus CNS is driven by subunit composition/stoichiometry as P2Xs were probed in the same expression system and recording conditions.

Studies of toluene action on voltage-gated ion channels are scarce. Acute exposure of neo-cortical cells in culture to submillimolar doses of toluene inhibits both peak and end of current mediated by voltage-dependent Ca^2+^ channels (VDCCs), being more efficacious on the latter. Likewise, acute submillimolar concentrations of toluene reversibly block VDCCs in differentiated PC12 cells, with inhibition of the end current being slightly more potent than inhibition of peak current, a difference hypothesized to be mediated by toluene’s ability to increase the VDCC inactivation rate (90). Toluene significantly shifts the activation of VDCCs, as well as their steady-state inactivation, in a hyperpolarizing direction. Remarkably, N-type VDCCs are more sensitive to toluene than the L type. Since the fourth transmembrane region of VDCC α subunits has highly conserved residues that sense transmembrane voltage, and transmembrane segments (particularly TM6) aid in inactivation, Shafer et al. (90) hypothesized that such regions play an important role in mediating toluene’s effects on VDCCs. They also speculated that these effects on VDCCs may contribute to the acute neurotoxicity caused by toluene.

Following expression in *X. laevis* oocytes, voltage- and Ca^2+^-gated K^+^ channels of the BK type (homomeric slo1 channels) are also inhibited by acute toluene, yet at concentrations greater than 1 mmol (91). Toluene does not change the voltage-activation curve, and it remains unknown whether toluene action on slo1 channels is dependent on the presence of the channel’s natural ligand, as reported for extracellular ligand-gated channels. Regarding inwardly rectifying K^+^ channels (Kirs), G protein–activated Kir (GirK) and Kir3.X, which are largely expressed in the brain tissue, are both inhibited by toluene in a subunit-specific manner. Moreover, while GirK2 is inhibited by 1 or 3 mmol of toluene, GirK1/2 and GirK1/4 are refractory. In the same expression system, voltage-gated Na^+^ channels (Na_v_) that are naturally abundant in skeletal muscle (Na_v_1.4) are also inhibited by millimolar concentrations of toluene (92). However, channel isoforms that are present in the brain are much more sensitive (IC_50_ = 0.3 mmol) (93, 94). Therefore, as reported for extracellular ligand-gated channels, the subunit composition of the ion channel complex is a key determinant of the response of voltage-gated ion channels to acute toluene. However, as with other ion channel families, the absence of structural biology data precludes us from knowing whether toluene-sensing ion channel subunits contain a toluene-binding site(s), an essential stepstone in a pharmacological strategy to counteract toluene actions in the brain via ion channel protein targeting.

### Protein Receptors Other Than Ion Channels

Studies on toluene actions on metabotropic receptors are restricted to G protein–coupled receptors (GPCRs). Thus, stimulatory responses to toluene can be blocked by remoxipride, a D2 antagonist (60). Repeated exposure to inhaled toluene produces behavioral sensitization (known to associate with substance use craving and relapse) (95), as measured by an enhanced locomotor response that is associated with DA transmission, yet in this case a decrease in D1 receptors in the PFC, NAc, hippocampus, and caudate is observed (96). Toluene-induced enhancement of intracranial self-stimulation, motor incoordination, memory impairment, social interaction deficits, and hyperlocomotion are reversed by LY379268, a metabotropic glutamate receptor agonist (97, 98). LY379268 could act on autoreceptors to decrease glutamate release, thus reducing stimulation of *N*-methyl-d-aspartate receptors (NMDARs). The mGluR5 antagonist 2-methyl-6-(phenylethynyl)pyridine decreases toluene-induced cognitive deficits and hypothermia, likely by reducing NMDAR stimulation (99). Thus, some toluene effects involving ionotropic glutamate receptors may involve metabotropic glutamate receptors.

In mice, acute inhalation of toluene (3,000–12,000 ppm) induces head twitches, resulting from 5-HT_2A/2C_ receptor stimulation (100). Moreover, atypical antipsychotics with 5-HT_2A_ antagonist or 5-HT_1A_ agonist properties reverse some cognitive and social interaction deficits caused by toluene (101, 102). Lastly, chronic toluene enhances the behavioral responses to hallucinogenic 2,5-dimethoxy-4-iodoamphetamine, which is thought to produce its effects via 5-HT_2A_ receptors (101).

Acute inhalation of toluene (4,000–12,000 ppm) significantly reduces μ-opioid receptor levels in several brain areas without impacting morphine-induced analgesia (103, 104), suggesting that other behaviors modulated by μ-opioid receptors may be impacted by toluene (105). Collectively, several studies have implicated GPCRs in some of the behavioral and functional effects of inhalant compounds such as toluene, although whether these receptors directly bind toluene is unknown.

## MOLECULAR MECHANISMS

### Modification of Protein Receptor Function Through Toluene-Membrane Lipid Interaction

The possibility that changes in receptor function/neurotoxicity caused by toluene result from an initial interaction between this volatile compound and membrane lipids is an early hypothesis that follows a predicament similar to that proposed for the neural basis of anesthesia by inhalational anesthetics (106): Anesthetic potency shows a strong correlation with the agent’s partitioning in cell membranes, which is in turn highly correlated with the anesthetic molecule’s overall hydrophobicity (Meyer–Overton rule) (107–109). The interpretation of such a correlation suffers from several setbacks, as discussed for anesthetics (110) and other hydrophobic ligands (111).

Support for membrane lipids as primary targets of toluene action in the brain stems from positive and negative findings: (*a*) the difference between the concentrations needed by neurotransmitters (e.g., GABA), neuromodulators (e.g., neurosteroids), and even exogenous agents (e.g., benzodiazepines/barbiturates) to alter receptor function (e.g., GABA_A_ receptors) through defined protein sites (nanomolar to micromolar range) versus toluene effective concentrations (greater than millimolar concentrations); and (*b*) the absence of structural information on docking sites for toluene in receptor proteins thought to mediate toluene actions on cell targets in the brain. While the location of general anesthetics in a lipid membrane is the bilayer hydrophobic region (107, 108), the precise membrane location for toluene, to our knowledge, remains undetermined. Assuming a similar location (based on hydrophobicity), such a location would likely cause membrane lipid disordering, as most general anesthetics do (107). This effect could be consequent to lipid peroxidation triggered by toluene’s presence (112). Another consequence of a highly hydrophobic molecule partitioning in the hydrophobic core of a cell membrane is a change in bilayer thickness, which would alter surface potentials and thus, voltage-dependent channel function (109). As discussed, however, intoxicating concentrations of toluene primarily disrupt ligand-gated ion channels, and the intrinsic excitability of neurons appears resilient. Mechanistic studies on the modification of bilayer thickness upon insertion of drugs differing in hydrophobicity have been conducted using the channel peptide gramicidin: Benzene (or desmethyl-toluene; Figure 1a) partitions into the bilayer hydrophobic core and thus modifies channel gating (113). Whether this mechanism applies to toluene action on oligomeric ion channel proteins remains to be determined. Of note, while hydrophobic volatiles increase membrane thickness, *N*-alkanols such as ethanol (Figure 1a) do not (109). Despite this difference, toluene and ethanol acute intoxications share most neurological signs and symptoms.

Protracted toluene exposure does lead to changes in lipid composition/arrangement within membranes. Thus, *Pseudomonas putida* tolerance to short-term toluene exposures involves the transformation of *cis*-9,10-methylene hexadecanoic acid to unsaturated 9-*cis*-hexadecenoic acid and then to its *trans* isomer. Longer toluene exposures additionally evoke a relative increase in cardiolipins and a decrease in phosphatidylethanolamines, changes that may increase lipid order and thus prevent further membrane partitioning of toluene (114). Increases in membrane order are found in volatile-tolerant thermophilic bacteria (115). In turn, *P. putida* mutant 32 has an unusually high amount of lipid species that increase its cell membrane surface hydrophobicity, which allows increased penetrance of toluene, making this mutant highly sensitive to this drug (116). When data from *P. putida* are compared to those from other bacteria, it becomes evident that while different gram-negative strains utilize different lipids to counteract toluene presence, the common end point is to maintain lipid order despite the continuous presence of toluene (117–119). Indeed, gram-negative bacteria grown in toluene are resilient to toluene’s membrane-disordering effect, as measured by changes in membrane polarization (120). However, *Staphylococcus haemolyticus*, a gram-positive bacteria, is able to tolerate 100% toluene and other BTEX compounds by increasing lipid species that decrease membrane order (121). Whether the change in membrane order plays a part in the differential toluene sensitivity across cells, brain regions, and length of exposure remains to be determined.

### Modification of Receptor Function Through Direct Docking of Toluene onto Protein Sites

Whether toluene modifies the activity of the receptors involved in toluene actions in the CNS (see previous section) by direct binding to these receptors remains unknown. Extrapolation of information from agents that share a given CNS effect with toluene is particularly difficult. For example, lysergic acid diethylamide (LSD), psilocybin, and mescaline (Figure 1b) have all been reported to evoke hallucinations via interaction with 5-HT_2A_ receptors (122, 123; reviewed in 124). Whether full agonists, partial agonists (e.g., LSD), or inverse agonists, binding of these ligands to 5-HT_2A_ receptors involves shared mechanisms, such as salt bridge formation between Asp155 and a positively charged nitrogen in each ligand (125). This and other interactions that involve polar groups make it unlikely that the simple and hydrophobic structure of toluene and related compounds (Figure 1a) could dock onto the 5-HT_2A_ binding site to which LSD and analogs bind. To our knowledge, there are no structural data documenting a direct docking of toluene molecules onto brain protein receptors in mammals. However, robust evidence demonstrates docking of toluene onto discrete areas in proteins of nonmammalian origin. Analysis of high-resolution images depicting toluene binding to nonmammalian species may reveal common patterns in the interactions between toluene molecules and specific amino acids. Moreover, dimensions of toluene-binding sites in nonmammalian species may be useful when searching for toluene-binding sites in mammalian protein species with known high-resolution structures. Thus, knowledge gained from detailed analysis of toluene-binding proteins in nonmammal species can potentially be used to explore the field of toluene protein targets in the mammalian brain. Search of 3D structures in the Protein Data Bank (PDB; https://www.rcsb.org) with the name “toluene” rendered 40 results as of April 2023: Only 6 contained toluene as a ligand as opposed to being a solvent. Analysis of these crystallographic data from bacterial/viral proteins unveils several features of toluene interactions with proteins, as follows.

**Figure 2:**
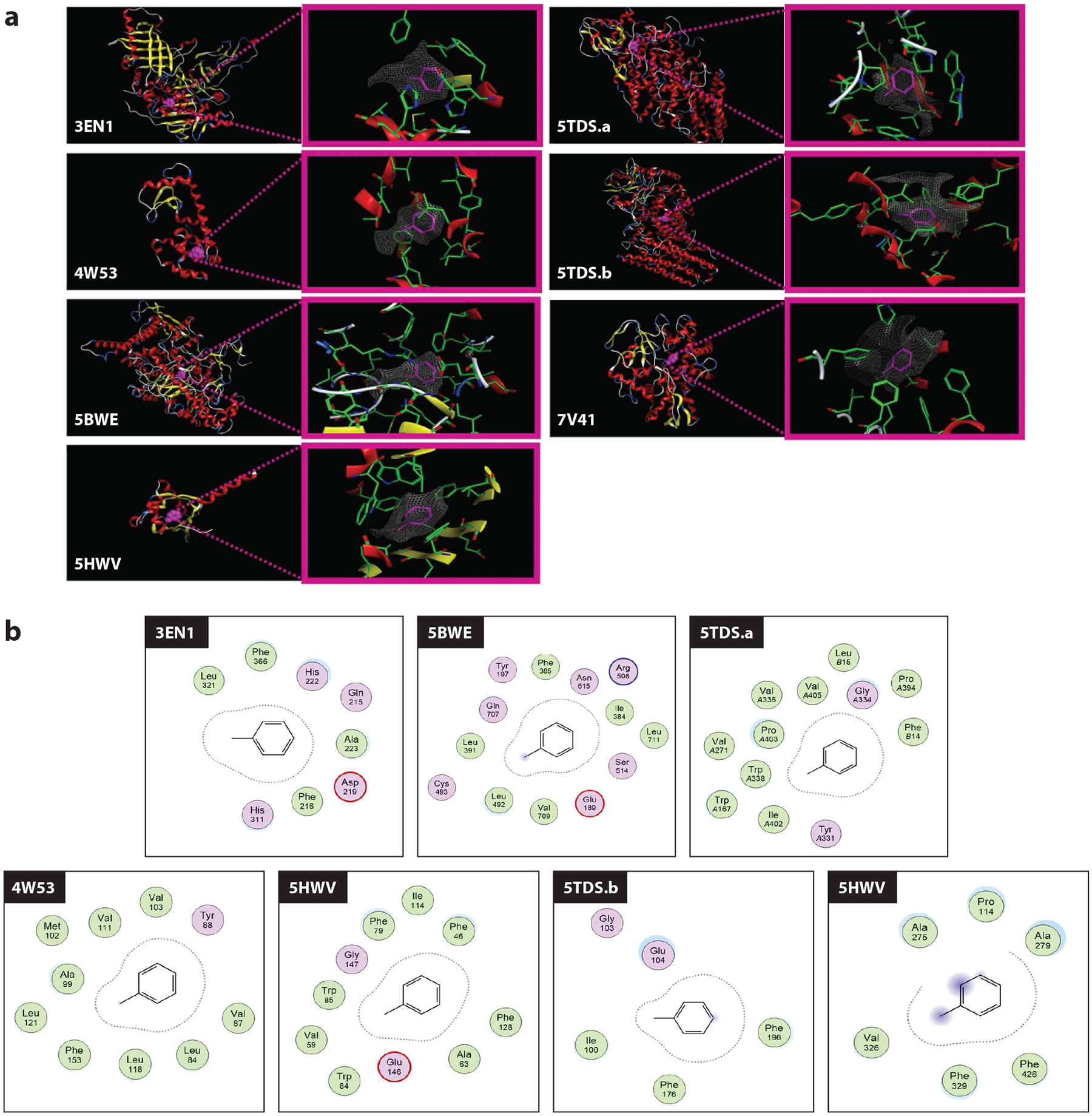
Toluene-binding sites on nonmammalian proteins may direct future research on the identification of toluene-binding sites in mammalian brain proteins. (*a*) Protein Data Bank (PDB) identification numbers for each high-resolution protein structure are shown in white. The following proteins are depicted: PDB IDs 3EN1 [toluene 2,3-dioxygenase (*Pseudomonas putida*); https://www.rcsb.org/structure/3en1], 4W53 [T4 lysozyme L99A (*Tequatrovirus T4*); https://www.rcsb.org/structure/4w53], 5BWE [chain A of benzylsuccinate synthase alpha-beta-gamma complex (*Thauera aromatica*); https://www.rcsb.org/structure/5bwe], 5HWV [TodS Per-Arnt-Sim 1 sensor domain (*Pseudomonas putida F1*); https://www.rcsb.org/structure/5hwv], 5TDS.a and b [toluene 4-monooxygenase (*Pseudomonas mendocina*); https://www.rcsb.org/structure/5TDS], and 7V41 [P450 monooxygenase (*Rhodococcus coprophilus*); https://www.rcsb.org/structure/7V41]. High-resolution structure data sets were uploaded and visualized using MOE2022.02 software (Chemical Computing Group, Canada). Alpha-helixes, beta-sheets, and disorganized protein regions are shown in red, yellow, and light gray, respectively. On panels with whole-protein views, toluene is depicted in pink. Inserts in red frames detail protein pockets that accommodate toluene. Surface maps of ligand-protein Van der Waals interactions are shown as gray nets. (*b*) 2D ligand-protein interaction maps derived using a built-in function in MOE2022.02. The distance cutoff between toluene and protein heavy atoms was set to 4 Å. PDB IDs are shown within black boxes at the top left corner of the corresponding ligand-protein site. Nonpolar and polar amino acids are depicted as green and lavender spheres, respectively. Charged amino acids are encircled in red lines. Three ligand-protein interaction maps point at toluene exposure to solvents (PDB IDs 5BWE, 5TDS.b, and 5HWV). Exposed areas involve both the phenyl ring and methyl group (*blue shading*). As somewhat expected, ligand-protein interaction maps reveal the predominant presence of aromatic (phenylalanine, tyrosine) and hydrophobic amino acids (alanine, valine, leucine) within 4 Å of toluene heavy atoms. While the handle of the toluene molecule is predominantly surrounded by valine, the vicinity of the toluene pan is enriched with aromatic phenylalanine. Overall, polar amino acids such as histidine, tyrosine, glutamic acid, and asparagine are scarce. Notably, threonine and hydrophobic methionine were absent in all maps.

One of the earliest high-resolution structures (PDB ID 3EN1; 126) describes toluene binding to the active site of toluene 2,3-dioxygenase from soil bacteria *P. putida*. In this structure, toluene resides between beta-sheets and alpha-helical domains of the protein (Figure 2a). The Van der Waals surface map follows the overall contour of toluene with its characteristic pan corresponding to the phenyl and its elongated handle formed by a methyl attached to the phenyl (Figure 2b). Likewise, PDB entries 5BWE (chain A of benzylsuccinate synthase alpha-beta-gamma complex from *Thauera aromatica*) and 5HWV (TodS Per-Arnt-Sim 1 sensor domain from *P. putida F1*) show toluene molecules between protein beta-sheets and alpha-helixes (Figure 2a). This propensity of toluene to localize within structured protein regions is further observed in PDB entries 4W53 (T4 lysozyme L99A from *Tequatrovirus T4*) and 5TDS (5TDS.b, toluene 4-monooxygenase from *Pseudomonas mendocina*) (Figure 2a). However, in these two entries, toluene prefers alpha-helical regions over beta-sheets. Disordered protein regions can also be found nearby toluene, as seen in the second binding site within toluene 4-monooxygenase (PDB ID 5TDS.a) and in PDB ID 7V41 (P450 monooxygenase from *Rhodococcus coprophilus*) (Figure 2a).

Ligand-protein interaction maps reveal the presence of aromatic (phenylalanine, tyrosine) and hydrophobic (alanine, valine, leucine) amino acids within 4 Å of toluene heavy atoms (Figure 2b). Surprisingly, polar amino acids, including histidine, glutamine and aspartic acid, are also found (Figure 2b). A more detailed analysis of the amino acid shows that the handle of toluene is predominantly surrounded by valine (18% of the amino acids within 4 Å of toluene heavy atoms in all structures) (Figure 2b). Valine is followed by tryptophan (14%), phenylalanine (11%), and leucine (11%). As somewhat expected, the vicinity of the toluene pan is enriched with aromatic phenylalanine (26%), followed by hydrophobic alanine (11%) and valine (11%) (Figure 2b). Overall, the total distribution of the residues surrounding toluene molecules shows a strong predominance of nonpolar phenylalanine (19%), valine (14%), and leucine (10%). While the presence of polar amino acids such as histidine, tyrosine, glutamic acid, and asparagine is detected in some toluene-accommodating protein pockets (PDB IDs 3EN1 and 5BWE), their presence is scarce in others (PDB IDs 4W53, 5TDS.a, and 5HWV). Notably, hydrophobic methionine and threonine are absent in all.

In summary, defined features of toluene-protein direct interactions include a propensity of toluene molecules to reside near structured protein domains, particularly near alpha-helixes. This propensity is remarkable as it was also found important for direct interaction between ethanol, a drug that disrupts several neuronal functions and behaviors affected by toluene (127–130), and ionotropic receptors that mediate alcohol actions in the body (discussed in 131). Parallelisms between common structural findings for ethanol-toluene and the behavioral effects of these drugs, however, should be taken with caution; toluene and ethanol clearly differ in their modulation of several voltage-gated K^+^ channels when expressed in the same membrane (91, 129). Although the available number of high-resolution structures is too low to derive quantitative predictions, our present analysis may build a foundation for future elucidation of candidate toluene binding sites on brain protein receptors that participate in toluene’s toxicity.

## CONCLUSIONS

As a compound that many people are occupationally or environmentally exposed to, as well as an easily accessible drug inhaled by disproportionately young and underprivileged populations with the intention of achieving a high, the importance of understanding the toxic properties of toluene and its biological targets cannot be understated. Toluene’s highly lipophilic structure plays a key part in its detrimental action on white matter structures in the brain. Thus, toluene-induced brain toxicity results from its targeting of neuronal and glial cells, in addition to a cerebrovascular component, yet the precise molecular targets that mediate toluene-induced brain intoxication remain unidentified. This uncertainty arises from the chemical structure of toluene, which allows it to easily partition in lipid membranes and alter their physicochemical properties, and modify the function of multiple protein receptors (especially neuronal ion channels, as documented in trying to explain the reinforcing properties of this chemical), and from the existence of a myriad of confounding factors, mainly contaminating chemicals and psychological/neurological comorbidities, which may impact the final brain toxicity of toluene. However, identification of the molecular targets, sites of action, and mechanisms mediating toluene toxicity on the brain is fundamental to designing a rational pharmacotherapeutic strategy that may revert or at least ameliorate the psychological and neurological impairments caused by toluene exposure.
